# Case Report: Invasive mucinous adenocarcinoma mimicking interstitial pneumonia with autoimmune features: diagnostic pitfalls and reflections

**DOI:** 10.3389/fimmu.2026.1958951

**Published:** 2026-09-08

**Authors:** Jianguo Tian, Guoqiang Li, Hongyan Zhang, Zhenzhen Ma

**Affiliations:** 1 Department of Medical Imaging, Shengli Oilfield Central Hospital, Dongying, Shandong, China; 2 Department of Pathology, Shengli Oilfield Central Hospital, Dongying, Shandong, China; 3 Department of Rheumatology and Immunology, Shengli Oilfield Central Hospital, Dongying, Shandong, China

**Keywords:** invasive mucinous adenocarcinoma, interstitial pneumonia with autoimmune features, diagnostic pitfall, bronchoalveolar lavage fluid, case report

## Abstract

**Background:**

Interstitial pneumonia with autoimmune features (IPAF) is an exclusionary diagnosis that requires the exclusion of alternative etiologies, including malignancy. Invasive mucinous adenocarcinoma (IMA) of the lung can radiologically and clinically mimic interstitial lung disease (ILD), and some lung cancer patients may present with non-specific autoantibody positivity, creating a diagnostic trap that may lead to misdiagnosis as IPAF.

**Methods:**

We retrospectively analyzed the clinical data, serology, imaging, bronchoalveolar lavage fluid (BALF) cytology, and pathology of a 78-year-old female patient, combined with a literature review.

**Results:**

The patient presented with cough and sputum production lasting two months. Chest CT showed diffuse bilateral high-density opacities with septal thickening. Pulmonary function tests revealed moderate diffusion impairment, and Velcro crackles were present on auscultation. Autoantibodies were positive for ANA (1:100, cytoplasmic granular pattern), AMA-M2 (161.75 U/mL), RF (78.2 IU/mL), and anti-CCP antibody (300 U/mL). Notably, all systemic inflammatory markers (ESR, CRP, and six cytokines) were normal. BALF and liquid-based cytology revealed no malignant cells. Rheumatology consultation favored a diagnosis of IPAF, and empirical methylprednisolone 40 mg/d was initiated. After one week, chest CT showed no resolution; mycophenolate mofetil and nintedanib were added. Despite standard anti-infective and immunosuppressive therapy, imaging remained unchanged and tumor markers remained persistently elevated. CT-guided percutaneous lung biopsy was performed, and histopathological examination confirmed invasive mucinous adenocarcinoma (CK7+, focal CK20+, TTF-1−, Napsin A−).

**Conclusion:**

In patients with suspected IPAF, the combination of normal inflammatory markers with other atypical features should prompt re-evaluation of the autoimmune etiology. The lepidic growth pattern of IMA can lead to false-negative BALF cytology; a negative result does not exclude malignancy. As an exclusionary diagnosis, IPAF must be established only after thorough exclusion of infection, malignancy, and other causes. Immunosuppressive therapy should not be initiated based solely on autoantibody positivity and ILD imaging.

## 1 Introduction

Interstitial lung disease (ILD) comprises a heterogeneous group of disorders characterized by inflammation and fibrosis of the alveolar walls. In 2015, the American Thoracic Society (ATS) and European Respiratory Society (ERS) proposed the classification of “interstitial pneumonia with autoimmune features” (IPAF) to encompass ILD patients with autoimmune clinical and/or serological features who do not yet meet the diagnostic criteria for a defined connective tissue disease (CTD) (1). The diagnosis of IPAF depends on characteristic features in at least two of three domains (clinical, serological, and morphological) and is fundamentally an exclusionary diagnosis. However, in clinical practice, the diagnosis of IPAF is often overdiagnosed. When patients present with autoantibody positivity, and diffuse interstitial lung imaging, rheumatologists tend to presume an autoimmune etiology and initiate glucocorticoids combined with immunosuppressants. This diagnostic inertia may obscure the true underlying cause and lead to delayed diagnosis and inappropriate immunosuppressive therapy.

Invasive mucinous adenocarcinoma (IMA) of the lung is a rare subtype of lung adenocarcinoma, accounting for approximately 2%–10% of all lung adenocarcinomas (2). Histologically, tumor cells are tall columnar or goblet-shaped with abundant intracytoplasmic mucin, growing in a lepidic pattern along alveolar walls and secreting large amounts of mucin that fill the alveolar spaces. Radiologically, IMA often presents as diffuse bilateral patchy opacities, ground-glass opacities, or interlobular septal thickening, making it easily misdiagnosed as pneumonia or ILD (3). Furthermore, some lung cancer patients may have non-specific autoantibody positivity, further complicating the differential diagnosis between IMA and IPAF. More problematically, the lepidic growth pattern of IMA means that tumor cells remain attached to the alveolar walls and do not readily shed into the bronchial lumen or alveolar lavage fluid, resulting in frequent false-negative BALF cytology and liquid-based thin-layer preparations, thereby deepening the diagnostic trap.

Herein, we report a case initially considered to represent IPAF but ultimately pathologically confirmed as pulmonary IMA. The unique aspects of this case are: (1) the patient’s inflammatory markers were completely normal, which was an unexpected finding inconsistent with active autoimmune inflammation; and (2) negative BALF cytology and liquid-based thin-layer smears contributed to delay in considering malignancy. This report aims to remind rheumatologists that in the diagnostic workup of IPAF, inflammatory markers and autoantibodies must be interpreted in concert, and the limitations of BALF cytology must be clearly recognized to avoid diagnostic delay.

## 2 Case presentation

### 2.1 General information

A 78-year-old female patient was admitted to the Department of Respiratory Medicine on June 13, 2026, with a two-month history of cough and sputum production. Two months prior, she developed cough and profuse white viscous sputum without obvious precipitating factors. There was no fever, chest tightness, dyspnea, hemoptysis, or chest pain. Chest computed tomography (CT) showed diffuse bilateral high-density opacities with interlobular septal thickening.

Past medical history: Type 2 diabetes mellitus for more than 20 years; cerebral hemorrhage more than 10 years ago (no sequelae); hypertension for more than 30 years; hyperlipidemia for more than 30 years. Ectopic pregnancy surgery 50 years ago. Hyperthyroidism diagnosed >20 years ago, currently in remission.

Personal and family history: No smoking or alcohol history. No exposure to occupational dust, asbestos, silica, or other hazardous substances. No history of keeping birds or pets. No definite drug or radiation exposure. Denied family history of genetic diseases.

### 2.2 Physical examination on admission

Temperature 36.0 °C, pulse 66 beats/min, respiration 22 breaths/min, blood pressure 152/80 mmHg, pulse oxygen saturation 92% on room air. The patient was conscious and alert. Jugular veins were not distended, and superficial lymph nodes were not palpable. Breath sounds were coarse bilaterally, with Velcro crackles audible at both lung bases. Heart rhythm was regular, with no pathological murmurs. Abdomen was soft, without tenderness or rebound tenderness. Mild bilateral lower extremity edema was present.

### 2.3 Admission diagnosis

1. Interstitial pneumonia; 2. Essential hypertension; 3. Type 2 diabetes mellitus; 4. Old cerebrovascular disease; 5. Hyperlipidemia.

### 2.4 Auxiliary examinations

#### 2.4.1 Laboratory tests

- Arterial blood gas (nasal cannula, 21% O_2_): pH 7.45, partial pressure of arterial oxygen (PaO_2_) 70.3 mmHg, partial pressure of arterial carbon dioxide (PaCO_2_) 37.6 mmHg.
- Complete blood count, B-type natriuretic peptide (BNP), creatine kinase, lactate dehydrogenase, cardiac troponin, coagulation profile, and D-dimer; preoperative infectious screening, including anti-hepatitis C virus antibody (Anti-HCV), anti- treponema pallidum antibody (Anti-TP), anti-human immunodeficiency virus antibody (Anti-HIV), and hepatitis C virus core antigen (HCV-cAg); lipid profile, stool analysis, and thyroid function were all unremarkable.
- Liver and kidney function: total protein (TP) 61.0 g/L, albumin (ALB) 39.8 g/L; alkaline phosphatase (ALP) and gamma-glutamyl transferase (GGT) were within normal range.
- Tumor markers: carcinoembryonic antigen (CEA) 19.7 ng/mL (elevated), cancer antigen 15-3 (CA15-3) 97.20 U/mL (elevated).
- Immunoglobulin: immunoglobulin E (IgE) 123.00 U/mL.
- Inflammatory markers: erythrocyte sedimentation rate (ESR) and C-reactive protein (CRP) were normal; six cytokines (IL-2, IL-4, IL-6, IL-10, TNF-α, IFN-γ) were all normal.
- Rheumatologic markers: rheumatoid factor (RF) 78.20 IU/mL (reference range 1–14 IU/mL), anti-cyclic citrullinated peptide (anti-CCP) antibody 300 U/mL (reference range 0–46 U/mL).
- Antinuclear antibody profile: antinuclear antibody (ANA) 1:100 positive (cytoplasmic granular pattern), anti-mitochondrial antibody M2 (AMA-M2) 161.75 U/mL (elevated); all other antibodies were negative. Vasculitis and autoimmune myositis antibody panels were negative.

#### 2.4.2 Electrocardiogram

Sinus bradycardia with irregular rhythm, premature atrial contractions, ST-T changes.

#### 2.4.3 Bronchoscopy (June 15, 2026)

**Figure 1:**
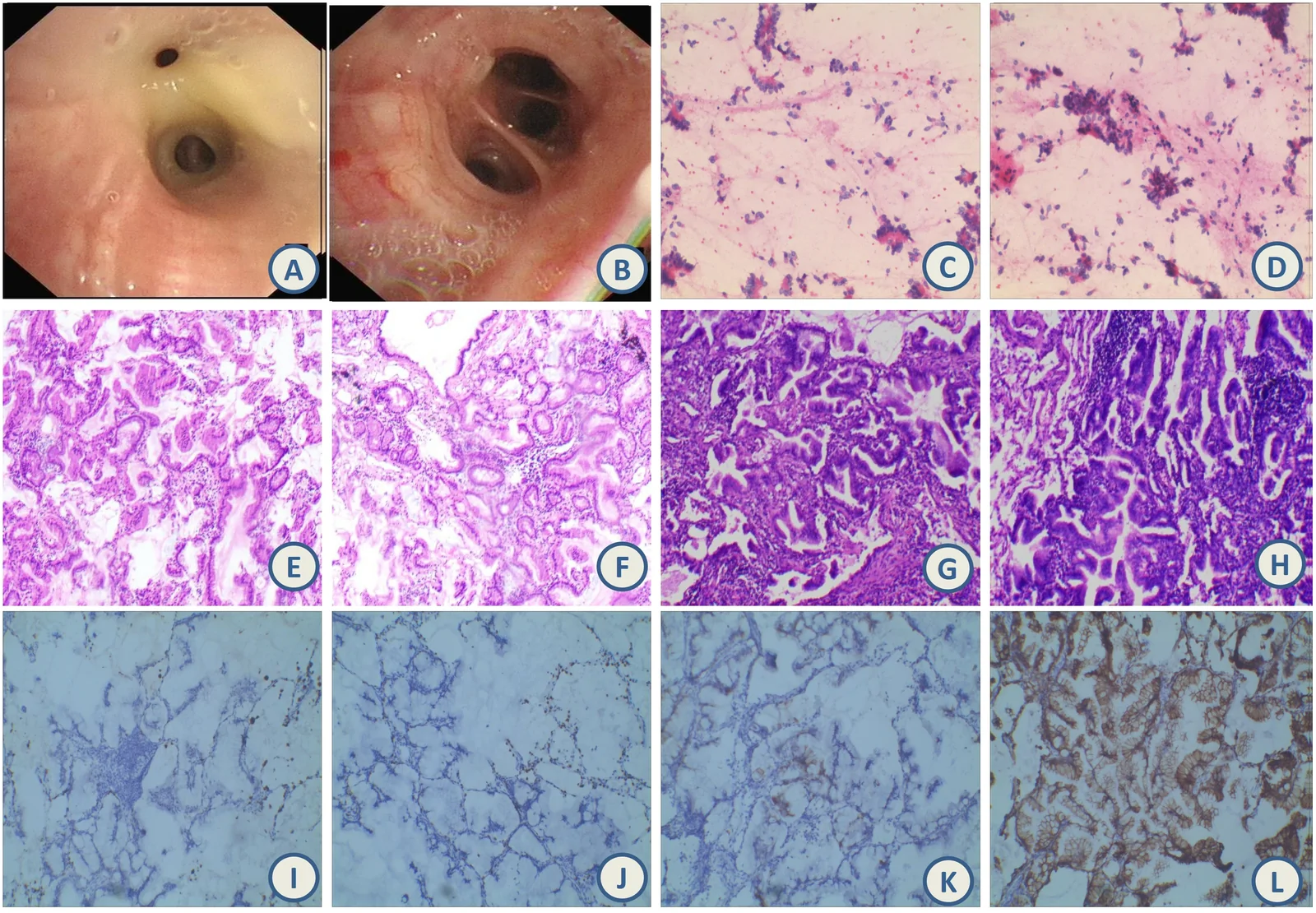
Bronchoscopy, cytology, and pathological examination. (June 15, 2026) Bronchoscopic examination and cytology: **(A)** Bronchoscopic view of the right lower lobe lateral basal segment, showing large amounts of viscous secretions almost completely obstructing the lumen. **(B)** Bronchoscopic view of the left lower lobe basal segment. **(C)** Brushing smear (right lower lobe) showing abundant bronchial glandular epithelial cells and a small number of lymphocytes; no malignant cells were identified. **(D)** Bronchoalveolar lavage fluid (BALF) and liquid-based thin-layer smear showing abundant bronchial glandular epithelial cells, macrophages, and a small number of lymphocytes; no malignant cells were identified. (July 6, 2026) Histopathology and immunohistochemistry of CT-guided percutaneous lung biopsy: **(E, F)** Hematoxylin and eosin (H&E) staining of lung tissue (×100) showing tumor cells growing in a lepidic pattern along alveolar walls. **(G, H)** H&E staining of lung tissue (×400) showing tumor cells with columnar/goblet-shaped morphology, abundant intracytoplasmic mucin, arranged in acinar and papillary patterns, with lumens filled with mucin. **(I)** Napsin A negative; **(J)** TTF-1 negative; **(K)** CK20 focally positive; **(L)** CK7 positive.

The trachea and visible segmental/lobar bronchi were patent, with mildly edematous mucosa. Large amounts of viscous secretions were seen in multiple segments; the right lower lobe lateral basal segment was almost completely obstructed by sputum (**Figures 1A, B**). Bronchoalveolar lavage (BAL) and brushing were performed in the right lower lobe basal segment and left lower lobe basal segment (**Figures 1C, D**). BALF cell differential: neutrophils 57%, macrophages 36%, lymphocytes 5%, eosinophils 2%. Microbiological examinations of BALF and brushings (bacteria, fungi, acid-fast bacilli) were all negative. BALF and liquid-based thin-layer cytology revealed no malignant cells. Transbronchial lung biopsy (TBLB) was not performed during this initial bronchoscopy. The decision was multifactorial: first, the patient was 78 years old with diffuse bilateral lung involvement, and the procedural risks—particularly pneumothorax and bleeding in the setting of compromised respiratory reserve—were considered significant; second, the diagnostic yield of TBLB for diffuse infiltrative disease is known to be limited, with a substantial false-negative rate; third, at this early stage, malignancy was not the leading diagnostic consideration given the NSIP-like imaging pattern and the absence of a discrete mass or nodule; fourth, the family expressed reluctance to proceed with invasive biopsy after being informed of the risks. In retrospect, this decision reflects the diagnostic pitfall central to this case—diagnostic anchoring to the IPAF hypothesis, combined with false reassurance from negative BALF cytology, led both the clinical team and the family to underestimate the necessity of early tissue acquisition.

#### 2.4.4 Imaging examinations

- Chest CT (June 13, 2026): Diffuse bilateral high-density opacities with interlobular septal thickening, suggestive of interstitial pneumonia; aortic and coronary atherosclerosis; splenic hilar calcification; intra- and extrahepatic biliary and pancreatic duct dilatation.
- Upper abdominal enhanced magnetic resonance (MR) + magnetic resonance cholangiopancreatography (MRCP) (June 18, 2026): Nodule in the pancreatic tail, considered splenic artery aneurysm with calcification; mild dilatation of intra- and extrahepatic bile ducts and pancreatic duct in the pancreatic body; bilateral pulmonary inflammation.
- Follow-up chest CT (June 22, 2026): Diffuse bilateral high-density opacities and interlobular septal thickening, essentially unchanged compared with the previous study.

#### 2.4.5 Pulmonary function tests (June 29, 2026)

Normal ventilatory function; moderate diffusion impairment (decreased DLCO, diffusing capacity of the lung for carbon monoxide).

### 2.5 Clinical course

**Figure 2:**
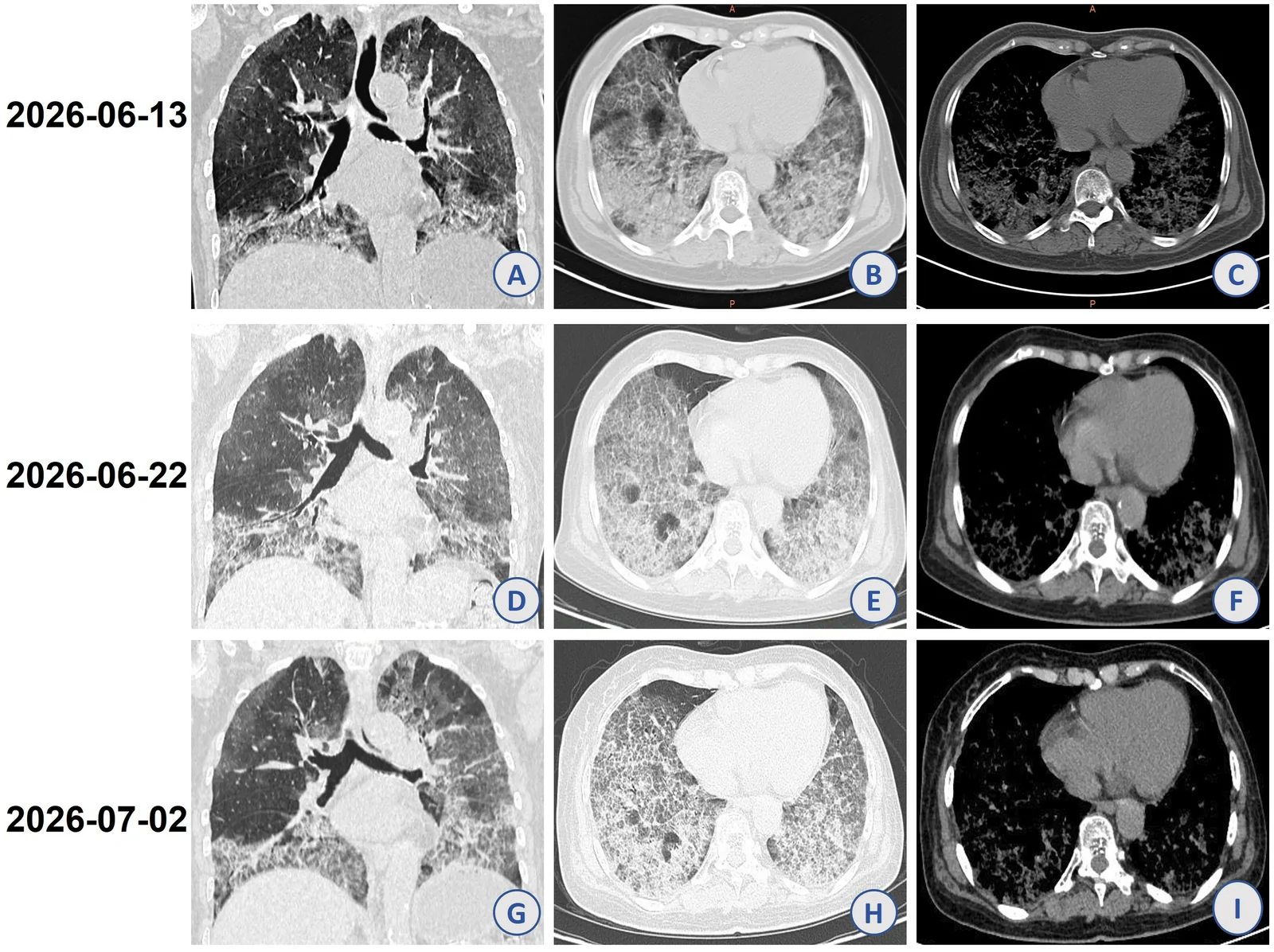
Evolution of chest CT imaging in the patient. Row 1 **(A–C)**: On admission (June 13, 2026), coronal view **(A)**, axial lung window **(B)**, and axial mediastinal window **(C)** showed diffuse bilateral high-density opacities with interlobular septal thickening. Row 2 **(D–F)**: Follow-up after one week of methylprednisolone 40 mg/d (June 22, 2026), coronal view **(D)**, axial lung window **(E)**, and axial mediastinal window **(F)** demonstrated no significant resolution of the bilateral lesions. Row 3 **(G–I)**: Re-examination after the addition of mycophenolate mofetil and nintedanib (July 2, 2026), coronal view **(G)**, axial lung window **(H)**, and axial mediastinal window **(I)** showed still no change in the pulmonary lesions.

**Figure 3:**
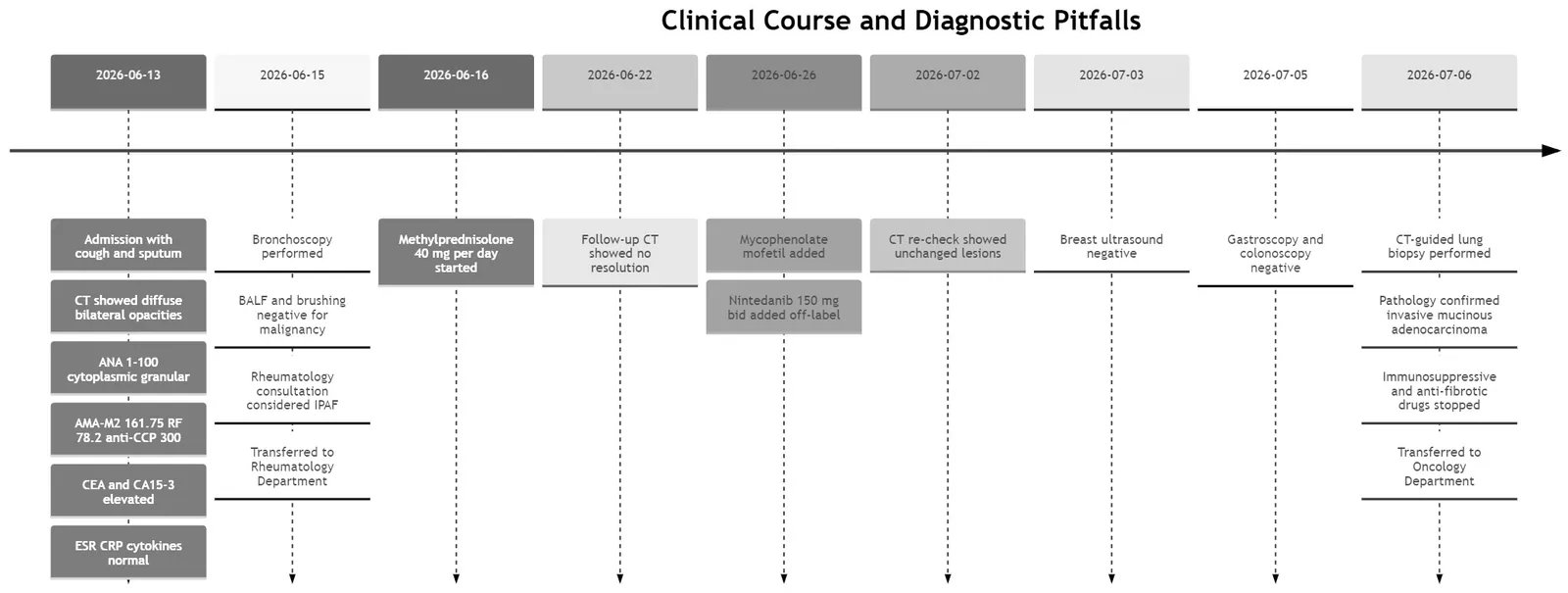
Clinical course and diagnostic pitfalls. Timeline summarizing the chronological sequence of diagnostic evaluations and therapeutic interventions from admission (June 13, 2026) to definitive diagnosis (July 6, 2026). Key events include: admission with diffuse bilateral opacities on CT and positive autoimmune serologies; bronchoscopy with BALF and brushing negative for malignancy, followed by rheumatology consultation considering interstitial pneumonia with autoimmune features (IPAF) and transfer to the Rheumatology Department; initiation of methylprednisolone 40 mg/day on June 16; follow-up CT on June 22 showing no resolution; addition of mycophenolate mofetil and off-label nintedanib 150 mg bid on June 26; CT re-check on July 2 demonstrating unchanged lesions; exclusion of extrapulmonary malignancy by negative breast ultrasound (July 3), gastroscopy, and colonoscopy (July 5); and CT-guided lung biopsy on July 6 confirming invasive mucinous adenocarcinoma, leading to discontinuation of immunosuppressive and anti-fibrotic drugs and transfer to the Oncology Department.

After admission, the patient initially received piperacillin-tazobactam combined with levofloxacin for anti-infection therapy, ambroxol for expectoration, diuresis, and management of underlying diseases. Rheumatology consultation on June 15, 2026, favored a diagnosis of IPAF based primarily on markedly elevated anti-CCP antibody (300 U/mL, reference range 0–46) and RF (78.20 IU/mL, reference range 1–14), both well above the ≥2× upper-limit-of-normal threshold for the serological domain, in conjunction with a non-specific interstitial pneumonia (NSIP)-like pattern on chest CT. Notably, this judgment was made despite the absence of articular manifestations (no synovitis, morning stiffness, or joint deformity), mucocutaneous features (no dry eyes, dry mouth, photosensitive rash, or oral ulcers), or myopathic features (no myalgia, muscle weakness, or mechanic’s hands). The patient also denied Raynaud’s phenomenon, skin tightening, or sclerodactyly. Empirical methylprednisolone 40 mg/d was initiated. Other findings, including ANA 1:100 (cytoplasmic granular pattern), AMA-M2 positivity, reduced DLCO, and Velcro crackles, were recorded as adjunctive features but were not individually decisive for the diagnosis. The patient was transferred to the Department of Rheumatology on June 16, 2026. After glucocorticoid therapy, the patient’s cough transiently improved, but follow-up chest CT on June 22, 2026, showed no resolution of the pulmonary lesions. On June 26, 2026, the treatment regimen was adjusted to add mycophenolate mofetil (MMF) 0.75 g twice daily and nintedanib 150 mg twice daily. On July 2, 2026, repeat chest and abdominal enhanced CT still showed no change in the pulmonary lesions (**Figure 2**). On July 3, 2026, breast ultrasound showed no obvious abnormality; on July 5, 2026, gastroscopy and colonoscopy showed no gastrointestinal tumors. Given that imaging showed no improvement after standard anti-infective and immunosuppressive therapy and tumor markers remained persistently elevated, a multidisciplinary discussion among rheumatology, radiology, and respiratory medicine was held, and CT-guided percutaneous lung biopsy was performed on July 6, 2026. Histopathological examination of the biopsy specimen confirmed pulmonary invasive mucinous adenocarcinoma. The tumor cells exhibited goblet or columnar morphology with abundant intracytoplasmic mucin, arranged in acinar and papillary patterns with mucin-filled lumens. Immunohistochemistry showed CK7 (+), focal CK20 (+), TTF-1 (−), Napsin A (−), and CK5/6 (−) (**Figures 1E–L**). Following pathological confirmation, methylprednisolone, MMF, and nintedanib were immediately discontinued. The patient was subsequently transferred to the Department of Oncology for further treatment (**Figure 3**).

## 3 Discussion

### 3.1 IPAF diagnostic criteria: conformity and departure in this case

The 2015 ATS/ERS IPAF diagnostic criteria require characteristic features in at least two of three domains (clinical, serological, and morphological) (1). This patient’s presentation demonstrated features that overlapped with two domains of the IPAF diagnostic framework:

- Serological domain: The patient exhibited markedly elevated anti-CCP antibody (300 U/mL, reference range 0–46 U/mL; >6× the upper limit of normal), which clearly fulfills the serological criterion of ≥2× upper limit of normal. RF was also elevated at 78.20 IU/mL (reference range 1–14 IU/mL; >5× the upper limit of normal), also fulfilling the threshold. Notably, the ANA titer of 1:100 with a cytoplasmic granular pattern does not fulfill the serological domain criteria for IPAF. The 2015 ATS/ERS IPAF research statement specifies an ANA titer of ≥1:320 on Hep-2 cells, or a defined nuclear staining (homogeneous, speckled, centromere, or nucleolar); cytoplasmic staining patterns are not included in the formal serological domain.
- Morphological domain: In the morphological domain, the chest CT demonstrated an NSIP-like pattern with diffuse bilateral ground-glass opacities and interlobular septal thickening, which qualifies as a characteristic morphological feature.

Strictly following the 2015 ATS/ERS criteria, although this patient demonstrated features consistent with the serological domain (RF, anti-CCP) and morphological domain (NSIP-like CT pattern), the most fundamental prerequisite of “excluding alternative etiologies” was not met. While features in two domains reached the required thresholds, a definitive IPAF classification could not be established because the exclusionary prerequisite—ruling out alternative etiologies such as malignancy—was not satisfied; consequently, the patient was incorporated into the IPAF treatment pathway through ‘relaxed criteria.’ Such diagnostic expansion is not uncommon in clinical practice and instead highlights the universal tendency of IPAF to be “over-diagnosed.” Moreover, the IPAF criteria explicitly state that the diagnosis must exclude other known etiologies, including drugs, environmental exposures, and malignancy (1). The lesson of this case is that when patients have autoantibody positivity and typical ILD imaging, clinicians readily regard IPAF as a “definite diagnosis” and initiate immunosuppressive therapy, ignoring the most fundamental exclusionary diagnostic principle.

### 3.2 From “mimicking ILD” to “mimicking IPAF”: upgraded diagnostic trap due to autoantibody positivity

Reports of lung adenocarcinoma (especially IMA) mimicking ILD are well-documented. In previous literature, IMA has been misdiagnosed as usual interstitial pneumonia (UIP), NSIP, or cryptogenic organizing pneumonia (COP) presenting with diffuse ground-glass opacities, interlobular septal thickening, Velcro crackles, and progressive dyspnea (4–11). The common feature of these cases is that the tumor closely mimicked the “morphological” manifestations of ILD in terms of imaging and symptoms, but was ultimately confirmed as mucinous adenocarcinoma by biopsy. A review of the existing literature reveals that the vast majority of IMA-mimicking-ILD cases did not involve autoantibody positivity (4–11). In other words, the diagnostic traps in these cases mainly were confined to the “imaging-pathology” level, and clinicians eventually discovered the tumor due to ineffective glucocorticoid therapy or the need for biopsy. The complexity of our case far exceeds that of previously reported cases: IMA not only mimicked the morphology of ILD but also mimicked the serological domain of IPAF. In 2023, Stevens et al. (12) reported a series of four cases at the CHEST conference describing four cases of TTF-1-negative mucinous adenocarcinoma with NSIP/OP-like imaging and concurrent autoantibody positivity (SSA, PM-Scl, MPO-ANCA), which were misdiagnosed as autoimmune-related ILD and treated with glucocorticoids. This represents one of the few reports involving autoantibody-positive IMA mimicking autoimmune lung disease. However, this series was a conference abstract with limited details, and it did not elaborate on the relationship between autoantibodies and inflammatory markers, the mechanism of false-negative BALF cytology, or the complete IPAF mistreatment course.

The unique value of our case lies in demonstrating that IMA can present with findings that overlap with two domains considered in the IPAF diagnostic framework: (1) serological domain—positive RF and anti-CCP antibodies; and (2) morphological domain—typical ILD imaging. However, IPAF is fundamentally an exclusionary classification, and an alternative etiology—ultimately identified as malignancy—had not been excluded. This “dual mimicry” led to the patient initially considered for the IPAF treatment pathway, receiving a combination of empiric therapies including glucocorticoids, immunosuppressants (MMF), and anti-fibrotic drugs (nintedanib), which has not been reported in detail in the existing literature. More importantly, this case reveals the heightened diagnostic risk posed by autoantibody positivity: when IMA only mimics ILD, the differential diagnosis of clinicians usually includes “infection vs. tumor vs. fibrosis”; however, when autoantibody positivity is added, diagnostic reasoning is readily constrained within the “autoimmune disease” framework, and malignancy is systematically deprioritized. In this case, the significant elevation of CEA and CA15–3 should have prompted priority consideration of pulmonary malignancy, but in the context of autoantibody positivity and negative BALF cytology, it was interpreted as “the need to exclude gastrointestinal/breast tumor” rather than “pulmonary tumor.” This diagnostic redirection caused by autoantibody positivity, combined with negative BALF cytology, may have contributed to the delayed consideration of malignancy in this case.

Therefore, this case is not only a reaffirmation of the known phenomenon that “IMA can mimic ILD” but also to the best of our knowledge, and based on our systematic literature search, the first detailed report of the rare and high-risk pattern that “IMA can mimic the presenting features considered in IPAF.” It reminds rheumatologists that when ILD patients have autoantibody positivity, diagnostic complexity does not diminish but rather increases substantially—because at this time, the differential must encompass not only CTD but also malignancy as a phenocopy of autoimmune disease.

### 3.3 Value and re-evaluation of inflammatory markers in the differential diagnosis of IPAF

Inflammatory markers (ESR, CRP, white blood cell and neutrophil counts, cytokine profiles, etc.) are important tools for evaluating autoimmune disease activity. In IPAF, the active alveolitis phase is usually accompanied by elevated ESR and CRP, and some patients may show increased pro-inflammatory cytokines such as IL-6 and TNF-α (13, 14). Abnormalities in these parameters reflect the active immune-mediated lung injury and are the pathophysiological rationale for initiating immunosuppressive therapy.

However, in clinical practice, the importance of inflammatory markers is often underestimated, especially when autoantibodies are positive; clinicians tend to focus on confirming an autoimmune etiology while overlooking the absence of inflammatory activity. The lessons from this case are as follows: (1) In this particular patient, the complete absence of systemic inflammatory activity represented a discordant finding that, in retrospect, should have prompted broadening of the differential diagnosis. It should be emphasized that normal inflammatory markers do not exclude IPAF or autoimmune-associated ILD, as some patients with established autoimmune ILD may have normal acute-phase reactants. However, when normal inflammatory markers coexist with advanced age, absence of systemic CTD manifestations, elevated tumor markers, and radiological abnormalities that persist despite empirical therapy, they may serve as a clue to reconsider the working diagnosis and broaden the differential beyond autoimmune etiologies. (2) In patients with ILD-like imaging but without evidence of systemic inflammation, the differential diagnosis should be broadened to include non-inflammatory etiologies. In addition to IMA, pulmonary alveolar proteinosis (15), pulmonary lymphangioleiomyomatosis (16), chronic eosinophilic pneumonia (inactive phase) (17), and other conditions can also present as diffuse lung disease with normal inflammatory markers. In elderly patients, exclusion of malignancy should always be the first priority. (3) The apparent dissociation between autoantibody positivity and normal inflammatory markers in this case suggests that these autoantibodies may not be the primary driver of the lung pathology. Studies have shown that the ANA positivity rate in people over 60 years old is as high as 22.7%, and the RF positivity rate is 6.5% (18); relying solely on autoantibody positivity to diagnose IPAF carries significant risk.

### 3.4 Limitations of BALF cytology in lepidic-pattern lung cancer: the key trap in this case

Early in the clinical course, bronchoscopy was performed. BALF cell differential showed neutrophil predominance (57%), microbiological examinations were negative, and BALF and liquid-based thin-layer smears revealed no malignant cells. This result strongly misled the clinical team—”malignancy has been excluded”—thus further biasing the diagnosis further toward IPAF. However, final pathology confirmed invasive mucinous adenocarcinoma. The core of this contradiction lies in the unique lepidic growth pattern of IMA.

The tumor cells of invasive mucinous adenocarcinoma arise from Clara cells or goblet cells of the bronchioles and exhibit lepidic growth along alveolar walls (2, 3). Tumor cells are attached to the alveolar wall surface in a single or multiple layers, protruding into the alveolar lumen, without disrupting the alveolar basement membrane or shedding in large numbers into the alveolar lumen. This growth pattern accounts for three key consequences: (1) An extremely low detection rate of tumor cells in BALF. Because tumor cells are tightly attached to the alveolar wall and do not readily shed into the alveolar lumen or bronchial lumen, BALF lavage and brushing fail to retrieve a sufficient number of tumor cells. Literature indicates that the positive rate of bronchial-derived cytology specimens (including BALF, brushings, and sputum) in lepidic-predominant adenocarcinoma is significantly lower than that in invasive non-mucinous adenocarcinoma or squamous cell carcinoma (19, 20). (2) Liquid-based thin-layer smears are susceptible to mucin-related dilution artifacts. IMA secretes large amounts of mucin, and BALF specimens are rich in mucin components, which presumably dilutes or obscures scattered tumor cells, leading to false-negative cytology. In this case, the BALF liquid-based smear only showed “bronchial glandular epithelial cells, macrophages, and a small number of lymphocytes,” which is the result of the combined effect of mucin interference and insufficient cell shedding. (3) Bronchoscopic brushing primarily samples intrabronchial lesions, whereas IMA primarily involves the alveolar spaces and bronchiolar walls in the early stage, rendering brushing poorly suited to sample the primary lesion.

Therefore, the negative BALF and liquid-based thin-layer smears in this case cannot be used as a basis for excluding lung cancer. Rather, in conjunction with the bronchoscopic finding of “large amounts of viscous secretions obstructing the lumen,” which is highly suggestive of mucinous adenocarcinoma, the possibility of false-negative cytology should be vigilantly entertained. For such patients, percutaneous lung biopsy or transbronchial lung biopsy (TBLB) should be actively pursued to obtain histological evidence.

### 3.5 Comprehensive mechanisms of IMA mimicking IPAF

In addition to the above two points, IMA mimicking IPAF involves the following mechanisms:

1. “Interstitial-like” imaging changes: IMA on CT often presents as diffuse bilateral ground-glass opacities, consolidations, or interlobular septal thickening in a “pneumonia-type” or “interstitial-type” distribution (3). Tumor cells grow along alveolar walls and secrete mucin, leading to alveolar wall thickening and lymphatic involvement in the interlobular septa, presenting on imaging as changes similar to pulmonary fibrosis or interstitial pneumonia.
2. Velcro crackles and diffusion impairment: The alveolar spaces are filled with mucin and tumor cells, leading to alveolar wall thickening and reduced gas exchange area, thereby producing Velcro crackles and moderate diffusion impairment. This pulmonary function change is similar to the UIP pattern.
3. Uncertain significance of autoantibody positivity: Regarding the ANA profile, the cytoplasmic granular pattern at a titer of 1:100 is most likely attributable to AMA-M2, given the strong association between anti-mitochondrial antibodies and cytoplasmic fluorescence on Hep-2 cells. The clinical significance of AMA-M2 positivity remains uncertain. Although the patient had no biochemical or radiological evidence of established primary biliary cholangitis at the time of evaluation, isolated AMA-M2 positivity may precede clinically overt disease and therefore cannot be attributed to the malignancy on the basis of this case. The ANA finding did not fulfill the formal IPAF serological-domain criterion, and its clinical significance in this patient remains uncertain. The markedly elevated RF and anti-CCP antibody in this patient raises several possibilities that cannot be distinguished from a single case: (a) coincidental or preclinical autoimmunity unrelated to the lung pathology; (b) autoantibody positivity occurring in the setting of malignancy. Lung adenocarcinoma, has been reported to induce polyclonal B-cell activation and non-specific autoantibody production, potentially driven by tumor-associated antigens, chronic inflammation in the tumor microenvironment, or aberrant citrullination of proteins by tumor cells (21, 22). Cases of lung cancer presenting with elevated RF, ANA, and even anti-CCP antibodies in the absence of autoimmune disease have been documented. The absence of a corresponding clinical phenotype makes an established CTD unlikely at the time of evaluation; however, it does not allow discrimination between preclinical autoimmunity, coincidental seropositivity, tumor-associated immune activation, and a paraneoplastic phenomenon. The true meaning of the autoimmune serology in this patient remains undetermined, and longitudinal rheumatological follow-up would be required to clarify whether these antibodies represent occult autoimmunity or tumor-related epiphenomena. Regardless of their origin, the key clinical lesson is that autoantibody positivity alone should not anchor the diagnosis to an autoimmune etiology when the overall clinical picture is atypical.
4. Warning value of tumor markers: At admission, CEA and CA15–3 were already significantly elevated, which was the earliest atypical feature warranting priority exclusion of malignancy. IMA has a strong correlation with CEA elevation (23), and CA15–3 can also be elevated in lung adenocarcinoma (24). For patients presenting with ILD-like imaging, significantly elevated tumor markers should prompt priority exclusion of lung cancer.

### 3.6 Treatment deviation and risks caused by misdiagnosis

Before the definitive diagnosis, this patient received nearly three weeks of glucocorticoid therapy (methylprednisolone up to 40 mg/d) and was once given MMF and nintedanib. This combination of empiric therapies carried multiple risks: (1) Glucocorticoids masking the condition: Glucocorticoids may transiently ameliorate symptoms through non-specific anti-inflammatory effects, potentially creating a transient apparent response that could delay definitive diagnostic procedures; (2) Potential tumor-promoting risk of immunosuppressants: MMF, as an immunosuppressant, may theoretically weaken the body’s immune surveillance against the tumor; (3) Nintedanib initiated empirically without documented progressive pulmonary fibrosis: Nintedanib was added on June 26, 2026, not because the patient fulfilled progressive pulmonary fibrosis (PPF) criteria-–she did not, as symptoms had transiently improved, pulmonary function had not been reassessed, and CT showed stability rather than progression-–but because the clinical team was alarmed by the extensive bilateral fibrotic-appearing infiltrates and elected to initiate anti-fibrotic therapy. In this patient, nintedanib was initiated empirically despite the absence of documented PPF and was discontinued after the diagnosis of malignancy. This illustrates how therapeutic escalation may occur before diagnostic clarification when ILD-like abnormalities are extensive. While nintedanib is not expected to treat the underlying neoplastic process and its use in this context may carry adverse effects and economic burden without anticipated benefit, antifibrotic therapy is preferably guided by an established fibrosing ILD phenotype and appropriate clinical criteria rather than by radiological severity alone.

Fortunately, percutaneous lung biopsy confirmed the diagnosis in a timely manner, avoiding prolonged inappropriate therapy and diagnostic delay. This experience profoundly illustrates that in the diagnostic workflow of IPAF, acquisition of pathological evidence should take priority over empirical immunosuppressive therapy.

### 3.7 Differential diagnostic implications for rheumatologists

Based on this case and literature review, we propose the following differential diagnostic strategies: (1) Comprehensive interpretation of inflammatory markers, autoantibodies, and imaging: In the diagnostic workup of suspected IPAF, inflammatory activity, autoantibody profile, and morphological findings should be evaluated together rather than in isolation. Normal inflammatory markers in the setting of atypical clinical features (advanced age, elevated tumor markers, or persistent radiological abnormalities despite empirical therapy) should prompt reconsideration of the working diagnosis and expansion of the differential to include non-autoimmune etiologies. (2) “IPAF” in elderly onset requires exclusion of malignancy first: In patients >70 years of age who present with pulmonary manifestations as the initial symptom and lack typical rheumatic clinical features, vigilance for lung cancer (especially IMA) is warranted. (3) Negative BALF cytology does not exclude lepidic-pattern lung cancer: For patients with large amounts of mucin secretion seen on bronchoscopy and diffuse ground-glass/consolidation imaging, negative BALF should be regarded as “indeterminate” rather than “excluded,” and tissue pathology should be actively obtained. (4) Lack of response to empirical glucocorticoid therapy should prompt reconsideration of the diagnosis and active pursuit of tissue pathology. It is important to note that treatment response is not a formal diagnostic criterion for IPAF, and the decision to obtain tissue sampling should not be delayed while awaiting a therapeutic trial when malignancy remains in the differential. (5) Clinicians should be vigilant against the tendency to overdiagnose IPAF: IPAF is an exclusionary diagnosis, but in clinical practice, the diagnosis is often established based solely on autoantibody positivity + ILD imaging. The 2015 ATS/ERS criteria should be strictly followed to avoid conflating “possible” or “incomplete” features with a definitive diagnosis.

### 3.8 Limitations

This study has several limitations inherent to its single-case design. First, the observations from this index case cannot be generalized as diagnostic rules. Normal inflammatory markers (ESR, CRP, and cytokines) do not exclude IPAF or autoimmune-associated ILD, and their value as a clue to reconsider the diagnosis requires validation in larger cohorts. Second, the etiology of the elevated anti-CCP antibody and RF—whether coincidental or preclinical autoimmunity, tumor-related epiphenomenon, or paraneoplastic process—remains undetermined from a single case, and no causal relationship between the malignancy and the observed autoantibody positivity can be established. The complete absence of CTD clinical features (no arthritis, sicca symptoms, myositis, sclerodactyly, or Raynaud’s phenomenon) argues against but does not exclude underlying autoimmunity. Third, the proposed mechanism for false-negative bronchoalveolar lavage fluid cytology (lepidic growth pattern combined with mucin dilution interference) is inferred from pathophysiological reasoning and the literature rather than directly demonstrated in this patient. Fourth, comprehensive molecular profiling of the tumor was not performed, limiting further insights into the biological behavior of invasive mucinous adenocarcinoma in this clinical context.

## 4 Conclusion

Pulmonary invasive mucinous adenocarcinoma can closely mimic IPAF. This case suggests that for patients with suspected IPAF, the combination of normal inflammatory markers with other atypical features (advanced age, elevated tumor markers, and lack of response to immunosuppression) should prompt re-evaluation of the autoimmune etiology. Furthermore, the lepidic growth pattern of IMA can lead to false-negative BALF cytology, and a negative result does not exclude malignancy. As an exclusionary diagnosis, IPAF must be established on the basis of thorough exclusion of infection, malignancy, and other etiologies. Immunosuppressive therapy should not be initiated based solely on autoantibody positivity and ILD imaging.

## Data Availability

The raw data supporting the conclusions of this article will be made available by the authors, without undue reservation.
